# Exposure to Heavy Metals and Serum Adiponectin Levels among Workers: A 2-Year Follow-Up Study

**DOI:** 10.3390/metabo13020158

**Published:** 2023-01-20

**Authors:** Chen-Jung Wu, A-Chuan Ho, Shih-Ya Chen, Chih-Hong Pan, Hsiao-Chi Chuang, Ching-Huang Lai

**Affiliations:** 1 Division of Family Medicine, Taoyuan Armed Forces General Hospital, Taoyuan 325, Taiwan; 2 Graduate Institute of Medical Sciences, National Defense Medical Center, Taipei 114, Taiwan; 3 School of Public Health, National Defense Medical Center, Taipei 114, Taiwan; 4 Institute of Labor, Occupational Safety and Health, Ministry of Labor, New Taipei City 221, Taiwan; 5 School of Respiratory Therapy, College of Medicine, Taipei Medical University, Taipei 110, Taiwan

**Keywords:** heavy metals, serum adiponectin, metabolic syndrome, lipid peroxidation

## Abstract

The workers exposed to metal fumes had an increased risk of metabolic syndrome, which was correlated with decreased serum adiponectin. Thus, we aimed to explore whether heavy metal exposure affects the adiponectin level. There were 96 male workers recruited from a shipyard at baseline. Apart from 82 participants completed the follow-up assessments, new participants were recruited in next year. Finally, there were 100 welding workers in the exposure group and 31 office workers in the control group. Inferential statistics on repeated measures were performed using generalized estimating equations. A weighted quantile sum (WQS) regression model was conducted to examine the joint effect of the multimetal exposure with serum adiponectin. Significantly negative associations of metals with adiponectin were detected in the welding workers, including Cr (β = −0.088; 95% CI: −0.148, −0.027), Mn (β = −0.174; 95% CI: −0.267, −0.081), Co (β = −0.094; 95% CI: −0.158, −0.029), Ni (β = −0.108; 95% CI: −0.208, −0.008), Cd (β = −0.067; 95% CI: −0.115, −0.018), and Pb (β = −0.089; 95% CI: −0.163, −0.015). The WQS regression suggested that Pb was the greatest contributor. In conclusion, our findings highlighted that welding workers exposed to heavy metals would reduce serum adiponectin.

## 1. Introduction

Welding is ubiquitous and indispensable in industrial development, and it consists of joining metals by fusing and melting them; it also includes tasks such as grinding, brazing, and soldering. It is estimated that more than 1 million workers worldwide perform welding as a part of their jobs [1]. Potential health hazards may arise during the welding process, including exposure to physical hazards, ergonomic stress, and chemical poisons. Welding fumes are a byproduct of the process, and the rate of generation and the composition of welding fumes are characteristic of the different materials being welded and the soldering components used [2]. Ultrafine and fine welding particles often consist of numerous metals, including iron (Fe), lead (Pb), zinc (Zn), nickel (Ni), copper (Cu), manganese (Mn), cobalt (Co), cadmium (Cd), and chromium (Cr) [3]. The size of the welding particles generated differs based on the industrial processes undertaken. Earlier studies have indicated that welding particles are in the ultrafine size range of 0.01 to 0.10 μm [4] and that most of the welding fumes have particles that are <0.50 μm in aerodynamic diameter [5]. Ultrafine particles have a higher likelihood of being deposited in the smaller branches of the bronchial airways in the respiratory tract; rapid clearance by the mucociliary system is much more ineffective. Generally speaking, exposure to heavy metals affects human beings by the routines of skin absorption, gut ingestion, and nasal inhalation of contaminated materials, varies from exposure frequency and intake quantity [6]. Heavy metals are regarded among the most persistent and hazardous pollutants found in the environment due to high toxicity and capacity to bioaccumulate even at low concentrations [7].

Few studies have shown that long-term heavy metal exposure could raise the risk of developing metabolic syndrome (MetS) [8], which is a constellation of cardiometabolic abnormalities and is recognized as a risk factor for cardiovascular disease and type 2 diabetes mellitus [9]. Additionally, a large cohort study that enrolled 10,059 male metal workers demonstrated that welding processed particles raised the risk of cardiovascular disease [10]. Previous studies have illustrated that the potential mechanisms by which metal fume exposure induces cardiometabolic illness might be correlated with systemic inflammation and the oxidative stress response [11]. 

Adiponectin is an adipose-derived hormone that is abundantly present in human plasma; it is adversely modulated by the accumulation of visceral fat and is lowered in obesity [12]. Adiponectin can increase insulin sensitivity in the skeletal muscle and the liver, and it is associated with reducing atherosclerosis [13]. Aside from these effects, adiponectin seems to display pleiotropic impacts on MetS. Emerging studies have emphasized the role played by hypoadiponectinemia in the pathogenesis of insulin resistance, MetS, diabetes, and cardiovascular disease [14,15]. Moreover, adiponectin also acts in the brain to enhance energy consumption and may thereby increase weight loss [16]. A large body of evidence indicates that adiponectin has a crucial role in the prevention of MetS [17]. Recent study even suggests that adiponectin might be an early marker of MetS that emerges before biochemical, anthropomorphic, and clinical parameters [18].

Given the awareness of the possible factors controlling adiponectin levels, including genetic, dietary, and lifestyle factors and environmental interventions, limited studies have discussed the relationship between occupational exposure to metals and adiponectin levels. Because welding workers are exposed to multiple heavy metals simultaneously, the combined effects of different heavy metals have not been discussed in previous surveys. By way of weighted quantile sum (WQS) regression, we could assess the weights of environmental chemicals, which allowed us to make clinical assumptions regarding relative chemical importance. Therefore, we designed a longitudinal study to explore the association between welding fume exposure and serum adiponectin levels.

## 2. Materials and Methods

### 2.1. Data Source and Participants

**Figure 1:**
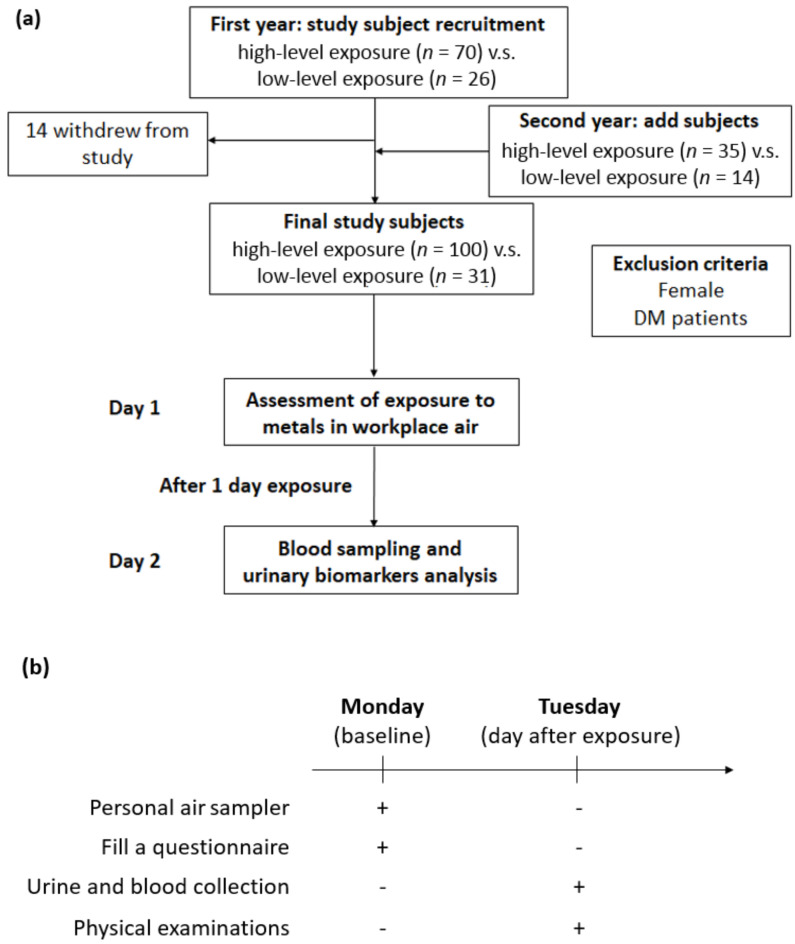
Experimental design for investigating the effects of metal fume fine on serum adiponectin in shipyard welders. (**a**) Flowchart of exposure assessment and subject recruitment. (**b**) Illustration of the experimental procedure for personal air sampler, urine collection, blood test, and physical examinations.

We recruited the study subjects from a shipyard in northern Taiwan, and all qualified subjects were classified into two groups based on their job responsibilities in 2014. At baseline, there were 70 welding workers in the exposure group and 26 office workers in the control group. The following year, earlier participants were recruited and underwent the same study protocols; moreover, we also included new study subjects in both the exposure and control groups. Fourteen subjects withdrew from the follow-up in 2015. Finally, a total of 131 study participants qualified for this study, namely, 100 welding workers and 31 office workers. In all, 82 subjects participated in 2 activities, and 49 subjects participated in one activity. Additionally, we excluded female subjects and individuals with diabetes or those receiving antidiabetic agents, as we intended to eliminate the impacts of sex and metabolism as much as possible. The associated descriptions were illustrated in Figure 1a.

All workers were asked to fill out a self-administered questionnaire that contained demographic and personal information, including medical history, respiratory protective devices used, and lifestyle habits. Furthermore, all study participants underwent physical examinations, morning urine sample collection after one day of exposure between 8 A.M. and 5 P.M. with 1 h of rest between 12 P.M. and 1 P.M. on Tuesday, and blood sampling after 8 h of overnight fasting. Additionally, all workers were required to wear personal air samplers for a 1-day exposure on Monday. The experimental procedure was shown in Figure 1b. The Ethics Committee of the Tri-Service General Hospital-Joint Institutional Review Board approved this research. All individuals in this survey provided written informed consent.

### 2.2. Measurement of Field Air Sampling

Personal air samples were collected by active samplers with filters of cellulose ester (pore size: 0.8 μm and diameter: 37 mm) from the workspaces of all participants; the active samplers had a flow rate of 2 L/min to gather the heavy metals. All workers were required to carry these personal samplers, which were placed in the breathing zone of the participants for a full eight-hour working day. The levels of heavy metals were detected using inductively coupled plasma–mass spectrometry (ICP–MS, Agilent 7500ce, Agilent, Santa Clara, CA, USA).

### 2.3. Assessment of Urinary Metal Concentrations

Morning urine samples were collected after one workday of exposure and stored at −80 °C until analysis. ICP–MS also measured the levels of urinary metals, and the procedures were performed twice as independent experiments. In addition, urine creatinine was measured by Jaffe’s method for adjusting urinary biomarker concentrations [19]. The median coefficients of variation of each metal were within 5–10%. The method detection limits for Cr, Mn, Co, Ni, Zn, Cd, Pd, and Cu were 0.0174, 0.0054, 0.0007, 0.0128, 0.0378, 0.0018, 0.0047, and 0.0275 ppb, respectively. For samples with metal concentrations below the limit of detection (LOD), a value equal to the LOD divided by the square root of 2 was assigned.

### 2.4. Measurement of Serum Adiponectin Levels

Fasting blood samples were collected in EDTA-treated tubes by venipuncture based on standard protocols, kept on ice, and delivered to our laboratory for further assessment. After centrifugation, fractions were separated and kept on ice, snap-frozen in liquid nitrogen, and stored at –80 °C until analysis. Serum adiponectin was analyzed with a commercial enzyme-linked immunosorbent kit (ELISA) (Calbiotech, Inc., El Cajon, CA, USA).

### 2.5. Measurement of Other Covariates

All participants underwent peripheral blood sampling after eight hours of overnight fasting to obtain cardiometabolic parameters, including triglycerides, total cholesterol, fasting glucose, serum albumin, and uric acid. Body mass index (BMI), expressed in kilograms by meters squared, was determined based on subjects’ height and weight. As previously described, a self-administered questionnaire was applied to acquire data on personal information and lifestyle factors.

### 2.6. Statistical Analysis

First, we used Shapiro-Wilk W test for examining normal data. Basic characteristics and biochemistry data between the control group and exposure group were compared using Student’s *t* tests for continuous variables if the variables were normal distribution. Otherwise, the Mann–Whitney test was conducted to evaluate the differences between these two groups. Discrete variables were examined by chi-squared tests. Since urinary metals and serum adiponectin were not normally distributed, log transformation was utilized to achieve a normal distribution. Generalized estimating equations (GEEs) on repeated measures were utilized to investigate the effectiveness of metal exposure in modulating serum adiponectin concentrations. GEEs with a first-order autoregressive working correlation matrix were conducted to model the study outcome while accounting for correlated data within the repeated measures of the study design. The changes in study outcome values from baseline to follow-up were expressed in the exposed and control groups under adjustment for potential confounding factors. The adjusted models compared both groups over time while accounting for age, BMI, serum albumin, total cholesterol, uric acid, fasting glucose, triglycerides, urine creatinine, job tenure, current smoking status, and exercise habits.

Moreover, a WQS regression model was conducted to examine the combined effects of the highly associated multimetal exposures with serum adiponectin. The WQS approach presumed that all metals had the same direction effects on the study outcome, regardless of positive or negative results [20]. A set of weights quantified the weights of the different influences of individual exposures. Each weight was confined within 0 and 1, and all the weights were summed to 1. WQS regression estimates were calculated from 1000 bootstraps with the sample separated into validation and training datasets by a split proportion of 4:6. Statistical analysis was performed using IBM SPSS statistics software for Windows version 22.0 (IBM Corp., Armonk, NY, USA). Additionally, WQS regression was implemented by means of the gWQS package for R 3.5.1. The definition of statistical significance was a two-sided *p* value of <0.05.

## 3. Results

### 3.1. Characteristics of the Subjects

**Figure 2:**
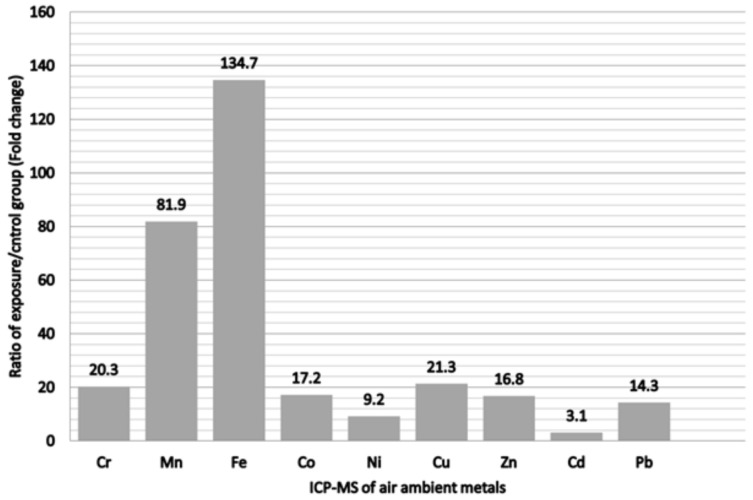
Ratio of metals of exposure/control group from personal air samples in the metal fumes particulate matter.

The ratios of the ambient air sampling of heavy metals for comparing the exposure and control groups are shown in Figure 2. Fe was the most abundant metal, which displayed a 134.7-fold change following exposure, followed by Mn in decreasing order with an 81.9-fold change. Others was followed by, in decreasing order, Cu, Cr, Co, Zn, and Pb. All metals in the ambient air sampling showed higher concentrations in the exposure group to varying degrees. The demographic characteristics of all participants are listed in Table 1. Compared with the office workers, the welding workers in this study were likely to be younger, had less job tenure, and had significantly higher blood albumin and urine creatinine levels. Nevertheless, these groups did not have significant differences in BMI, total cholesterol, triglycerides, uric acid, fasting glucose, or personal habits. In addition, all welding workers had used respiratory protective devices at work by self-report (data not shown). The comparison of urinary metals in the office and welding workers is shown in Table 2. Although there were no significant differences between the groups for all urinary metals, the concentration of urinary metals in the welding workers seemed to be higher than that in office workers.

### 3.2. Correlations of Urinary Metal Levels with Serum Adiponectin

The GEEs results for the correlations among metal exposure and serum adiponectin are shown in Table 3. After full adjustment for all variables, significantly negative associations of specific urinary metal levels with serum adiponectin concentration were detected among the welding workers, including Cr (β = −0.088; 95% CI: −0.148, −0.027, *p* < 0.01), Mn (β = −0.174; 95% CI: −0.267, −0.081, *p* < 0.001), Co (β = −0.094; 95% CI: −0.158, −0.029, *p* < 0.01), Ni (β = −0.108; 95% CI: −0.208, −0.008, *p* < 0.05), Cd (β = −0.067; 95% CI: −0.115, −0.018, *p* < 0.01), and Pb (β = −0.089; 95% CI: −0.163, −0.015, *p* < 0.05). However, there were no significant associations between urinary metals and serum adiponectin among office workers. Taken together, these findings indicated that occupational exposure to specific metals might have an impact on the reduction in serum adiponectin levels.

### 3.3. The Contributions of Multiple Metal Exposures to Serum Adiponectin

**Figure 3:**
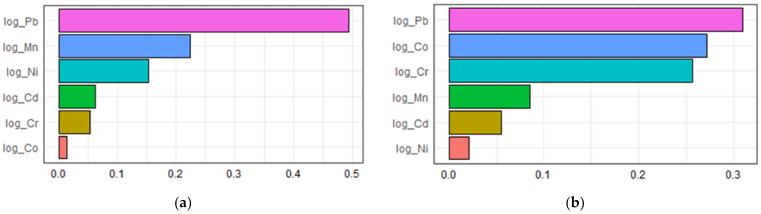
The associations between urinary metal levels and serum adiponectin levels based on weighted quantile sum regression analysis (**a**) unadjusted; (**b**) adjusted.

An estimate of −0.0435 (*p* = 0.048) per WQS unit in the unadjusted model and an estimate of −0.0478 (*p* = 0.026) per WQS unit in the adjusted model in the inverse correlation were shown in Table 4. The contributions of multiple urinary metal levels to serum adiponectin levels individually by the WQS regression model are illustrated in Figure 3. As displayed in Figure 3a, the highest contributors to serum adiponectin were Pb, making up 49.4% of the total contribution in the unadjusted model. After adjusting for confounders in the WQS regression model, an inverse association was found between urinary heavy metals and serum adiponectin. Figure 3b shows that Pb, Co, and Cr were 30.9%, 27.2%, and 25.7% contributors to serum adiponectin, respectively. Collectively speaking, regardless of these unadjusted or adjusted WQS regression models, among metal exposures, Pb was the most negative contributor and had the greatest influence on serum adiponectin levels.

## 4. Discussion

In this two-year follow-up study, the most prominent finding was that occupational exposure to specific metals was negatively linked to serum adiponectin levels in welding workers. Additionally, Pb was the most negative contributor to serum adiponectin levels. To the best of our knowledge, this study was the first to examine the impacts of occupational metal exposure on serum adiponectin levels using GEEs and WQS regression models.

An earlier study reported occupational and environmental exposure to Pb as a probable risk factor for cardiovascular disease [21]. Recent experimental and epidemiological studies have indicated that heavy metal exposure was considered a risk factor for cardiovascular disease and raised the public health burden [22,23]. These review articles discussed the potential correlation between chronic heavy metal exposure—including exposure to Pb, Cd, mercury (Hg), and arsenic (As)—and cardiovascular disease, although the mechanism through which heavy metals act to elevate cardiovascular risks remains disputed. These nonessential heavy metals were all nonthreshold toxins and could display toxic effects at trace concentrations [24]. A review article by Xu et al. proposed that exposure to increased levels of As, Cd, Pb, and Hg was significantly correlated with MetS or comorbid conditions [25]. Another cross-sectional study analysis derived from the Korea National Health and Nutrition Examination Survey of 1405 subjects indicated that a higher prevalence of MetS was correlated with increased blood Pb concentrations in Koreans [26]. Additionally, a prospective cohort study that enrolled 2500 young adults of African descent reported that blood As and Pb were significantly associated with elevated fasting glucose with adjustment for percent body fat [27]. In 2000, a review article that contained cell, animal, and human study results suggested the damaging role of Cd in the organic impairment of glucose metabolism; therefore, it contributed to insulin resistance [28].

However, limited literature has explored the relationship between heavy metals and adiponectin levels, especially in occupational exposure. An animal model executed by Kawakami et al. revealed that Cd exposure caused abnormal adipocyte differentiation, expansion, and function, which lowered the gene expression levels of adiponectin and might contribute to insulin insensitivity [29]. In 2013, the same study group designed another mouse model with Cd administration, which also indicated that Cd exposure induced abnormally smaller adipocytes and decreased adiponectin levels [30]. Another mouse model by the same author found that in vivo exposure to inorganic Co might exhibit a protective function in obesity-related diseases by increasing the adiponectin mRNA expression level in adipocytes and plasma adiponectin level. [31]. This finding was inconsistent with our study results. Apart from the animal studies, a longitudinal study by Wang and his colleagues that enrolled 1228 midlife women without specific heavy metal exposure illustrated that exposure to Cd was associated with an adverse adiponectin profile [32]. In line with earlier reports, our study results revealed that Cd exposure was correlated with decreased serum adiponectin levels, while Co exposure also demonstrated a negative adiponectin profile.

Our preceding study reported that Fe, Zn, Mn, and Cu were dominant among the welding fumes in a shipyard and that welding workers had higher urinary concentrations of Co, Cu, Ni, Mn, Cd, and Zn [33]. Thus, we selected the eight metals to test for in the postexposure urinary samples of the participants in this survey. Among these heavy metal exposures, Pb had the most negative influence on serum adiponectin levels according to the WQS model. One of the mechanisms was probably related to lipid disturbance in occupational Pb exposure. An earlier study by Ademuyiwa et al. suggested that Pb exposure increased cholesterol synthesis and transport to peripheral tissues among petrol station workers [34]. Aside from conventional mechanisms, telomere shortening and lipid disturbance were also regarded as unignorable roles in the pathway whereby low-level Pb exposure contributed to cardiovascular disease [35]. Moreover, a cross-sectional study of 986 subjects by Sirivarasai et al. indicated that exposure to low Pb levels correlated with deficiency of the enzyme catalase and oxidative stress, which might lead to high blood pressure [36]. In addition to toxic heavy metals, occupational exposure to some essential metals, such as Co, Ni, Cr, and Mn, also negatively affected serum adiponectin levels in our study. Actually, long-standing overexposure to and deficiencies in trace micronutrients could cause adverse health outcomes. Exposure to Mn, Ni, Cr, and Co has increased because of their use as industrial metals in commercial applications over the last century. A recent study summarized that the main detrimental health impact of Mn, Ni, and Co, to a lesser extent, was on lipid peroxidation arising from oxidative stress [37]. Cr had multiple oxidation states ranging from −2 to + 6, in which the trivalent and hexavalent forms were primarily stable structures. Cr(VI) was connected with toxicity and carcinogenicity, while Cr(III) was essential in trace amounts for protein and lipid metabolism and acted as a cofactor for insulin action [38]. Similar to other metals, Cr(VI)-induced oxidative stress and reactive oxygen species production at high concentrations affected the lipid content and DNA of cells, which resulted in lipid peroxidation and DNA damage, respectively [39].

Some limitations of this study should be considered. First, all welding workers were asked to use respiratory protective devices at work, but office workers were not compelled to wear masks whey they were in the shipyard. Therefore, some office workers might have contacted metal fumes during their workdays if they walked through the welding sites; this might have increased the exposure levels to heavy metals among the office workers more than we expected. Second, to eliminate sampling bias, we confined the work experience of office workers to those who had not held welding jobs within the past two years. However, some office workers in the shipyard had worked in the welding department when they were younger; therefore, the potential effects of earlier exposures to heavy metals on serum adiponectin levels could not be thoroughly assessed, although we corrected the covariates of job tenures. Next, some heavy metals, such as Cr, had multiple oxidation states, but urinary metals analyzed in this study by ICP–MS could not distinguish the amounts of individual oxidation states. Additionally, the welding aerosol composition varied depending on the specific type of welding process and materials used. Nevertheless, the work content of welding workers in this study could not be set up as a uniform welding process. Last, we did not include individual food intake or record dietary recall as confounding variables in this study. However, almost all workers obtained their lunch from factory-provided meals, which diminished the individual differences in the food consumption of the study subjects. Future works should be established with the basis to eliminate the shortcomings of study designs, including combination of high performance liquid chromatography and ICP-MS for analysis of oxidation states, adding dietary questionnaires, and setting up a standard welding process.

## 5. Conclusions

Adiponectin might be an early marker of MetS, and long-term heavy metal exposure could raise the risk of MetS. Our study findings, for the first time, suggest that occupational exposure to heavy metals including Cr, Mn, Co, Ni, Cd, and Pb decreases serum adiponectin levels in welding workers. Furthermore, Pb acted as the highest adverse contributor to lower adiponectin levels among metal exposures. Thus, we highlighted an important issue in the prevention of cardiometabolic diseases and occupational illnesses that de-serves public attention. In the future, more basic research is needed to clarify the disease pathology and cellular and molecular pathways involved.

## Figures and Tables

**Table 1:** Characteristics of welding and office workers in 2-year follow-up.

	Total Workers (n = 96)	Welding Workers (n = 70)	Office Workers (n = 26)	*p* Value	Total Workers (n = 131)	Welding Workers (n = 100)	Office Workers (n = 31)	*p* Value
	First Year				Second Year			
Continuous variables ^a^								
**Age** **(years)**	46.43 (12.04)	44.08 (11.15)	52.74 (12.28)	0.001	46.23 (12.80)	44.66 (12.39)	51.29 (12.97)	0.011
**BMI** **(kg/m^2^)**	25.21 (3.33)	25.15 (3.37)	25.36 (3.27)	0.784	25.25 (3.27)	25.11 (3.29)	25.70 (3.23)	0.381
**Albumin** **(g/dL)**	4.71 (0.21)	4.74 (0.21)	4.62 (0.19)	0.011	4.60 (0.23)	4.62 (0.22)	4.51 (0.24)	0.015
**TC** **(mg/dL)**	190.40 (56.77)	192.79 (64.07)	183.96 (29.33)	0.501	191.04 (55.96)	192.47 (61.69)	186.42 (31.35)	0.601
**TG** **(mg/dL)**	144.73 (201.03)	150.66 (231.96)	128.77 (68.58)	0.638	140.24 (187.07)	145.92 (211.42)	121.94 (60.79)	0.535
**UA** **(mg/dL)**	6.69 (1.50)	6.68 (1.56)	6.70 (1.32)	0.952	6.44 (1.25)	6.38 (1.22)	6.59 (1.35)	0.469
**FPG** **(mg/dL)**	94.55 (8.75)	94.56 (9.32)	94.54 (7.15)	0.993	96.04 (9.44)	96.07 (10.06)	95.94 (7.25)	0.945
**UC** **(mg/dL)**	133.82 (72.36)	143.53 (76.32)	107.70 (53.36)	0.030	122.77 (62.68)	128.92 (65.51)	102.93 (48.30)	0.043
**Job tenure** **(years)**	22.75 (15.40)	20.75 (15.28)	28.12 (14.70)	0.037	22.34 (16.63)	20.88 (16.61)	26.92 (16.11)	0.083
**Adiponectin** **(ug/dL)**	15.06 (8.73)	14.76 (8.02)	15.17 (9.04)	0.841	13.69 (6.93)	13.55 (7.30)	14.16 (5.62)	0.666
Categorical variables ^b^								
**Current** **Smoking**	49 (51.0)	39 (55.7)	10 (38.5)	0.170	75 (57.8)	62 (62.0)	13 (41.9)	0.062
**Current** **Exercise**	26 (27.1)	17 (24.3)	9 (34.6)	0.315	34 (26.0)	24 (24.0)	10 (32.3)	0.359

BMI, body mass index; TC, total cholesterol; TG, triglyceride; UA, uric acid; FPG, fasting plasma glucose; UC, urine creatinine; ^a^ Values were expressed as mean (standard deviation); ^b^ Values in the categorical variables were expressed as number (%).

**Table 2:** The concentration of urinary metals in welding and office workers in 2-year follow-up.

Urinary Metals (μg/L)		Total Workers	Welding Workers	Office Workers	*p* Value
Continuous Variables [GM (GSD)] ^a^					
**Urine Cr**	First year	3.07 (1.59)	3.22 (2.70)	2.73 (1.66)	0.205
	Second year	2.65 (1.35)	2.66 (1.30)	2.62 (1.33)	0.578
**Urine Mn**	First year	2.13 (2.21)	2.24 (2.42)	1.90 (2.16)	0.132
	Second year	2.89 (1.84)	2.94 (1.85)	2.75 (1.73)	0.621
**Urine Co**	First year	0.61 (2.05)	0.66 (2.13)	0.59 (1.66)	0.308
	Second year	0.66 (1.29)	0.75 (1.27)	0.62 (1.21)	0.675
**Urine Ni**	First year	10.92 (1.71)	11.18 (1.46)	10.32 (1.74)	0.279
	Second year	26.67 (1.82)	28.77 (1.71)	25.91 (1.75)	0.599
**Urine Zn**	First year	484.27 (2.00)	515.39 (1.85)	415.50 (2.00)	0.139
	Second year	472.98 (1.64)	491.55 (1.78)	427.91 (1.83)	0.408
**Urine Cd**	First year	0.62 (1.82)	0.64 (1.93)	0.56 (1.76)	0.504
	Second year	0.64 (1.75)	0.65 (1.31)	0.62 (1.69)	0.571
**Urine Pb**	First year	6.17 (2.88)	6.29 (3.14)	5.87 (2.64)	0.421
	Second year	31.95 (1.26)	32.26 (1.22)	30.95 (1.27)	0.360
**Urine Cu**	First year	105.93 (2.02)	108.44 (1.54)	100.00 (2.10)	0.242
	Second year	119.95 (2.16)	125.25 (2.21)	117.97 (2.03)	0.703

GM, geometric mean; GSD, geometric standard deviation; ^a^ Mann-Whitney U test.

**Table 3:** Association between urinary metal concentrations and serum adiponectin levels by GEEs.

Exposure Markers (log μg/L)	Welding Workers		Office Workers	
	Serum Adiponectin (log μg/mL) ^a^			
	β ^b^ (95% CI)	*p* Value	β ^b^ (95% CI)	*p* Value
**Urine Cr**	−0.088 (−0.148, −0.027)	0.004	0.022 (−0.088, 0.133)	0.695
**Urine Mn**	−0.174 (−0.267, −0.081)	<0.001	0.020 (−0.116, 0.156)	0.776
**Urine Co**	−0.094 (−0.158, −0.029)	0.004	0.017 (−0.082, 0.116)	0.731
**Urine Ni**	−0.108 (−0.208, −0.008)	0.033	−0.004 (−0.157, 0.148)	0.956
**Urine Zn**	−0.095 (−0.213, 0.022)	0.112	−0.030 (−0.173, 0.112)	0.677
**Urine Cd**	−0.067 (−0.115, −0.018)	0.007	0.007 (−0.068, 0.083)	0.850
**Urine Pb**	−0.089 (−0.163, −0.015)	0.018	0.029 (−0.068, 0.126)	0.554
**Urine Cu**	−0.025 (−0.214, 0.165)	0.800	−0.127 (−0.273, 0.527)	0.533

^a^ Adjusted covariates including age, BMI, serum albumin, total cholesterol, uric acid, fasting glucose, triglyceride, urine creatinine, job tenure, current smoking, current exercise. ^b^ β coefficient was interpreted as the change of log(adiponectin) level for each by a one unit increased in log(urinary metal concentration).

**Table 4:** β coefficients from the weighted quantile sum regressions for serum adiponectin levels.

	β Coefficients ^a^	*p* Value	Weight (%)					
			log Pb	log Mn	log Ni	log Cd	log Cr	log Co
Unadjusted	−0.0435	0.048	49.4	22.4	15.3	6.3	5.3	1.4
Adjusted ^b^	−0.0478	0.026	30.9	8.6	2.1	5.5	25.7	27.2

^a^ β coefficients are per increase of an interquartile range of the logarithmically transformed urinary metals. ^b^ Variables adjusted for include age, BMI, and job tenures.

## Data Availability

The datasets used and/or analyzed during the current study are available from the corresponding author on reasonable request. The data are not publicly available due to personal information protection.
